# Relationship between feeding milestones and comorbidities with the postnatal growth in preterm infants

**DOI:** 10.1038/s41372-026-02744-4

**Published:** 2026-06-09

**Authors:** Faith Bala, Enas Alshaikh, Sudarshan R. Jadcherla

**Affiliations:** 1 The Innovative Infant Feeding Disorders Research Program, Nationwide Children’s Hospital, Columbus, Oh USA; 2 Center for Perinatal Research, The Research Institute at Nationwide Children’s Hospital, Columbus, Oh USA; 3 Division of Neonatology, Nationwide Children’s Hospital, Columbus, Oh USA; 4 Division of Pediatric Gastroenterology, Hepatology, and Nutrition, Department of Pediatrics, The Ohio State University College of Medicine, Columbus, Oh USA

**Keywords:** Outcomes research, Risk factors

## Abstract

**Objective:**

To examine the relationship between feeding milestones and the postnatal growth of preterm infants.

**Study design:**

Retrospective study of 77 infants born <32 weeks’ gestation. Growth variables at birth and discharge were converted to age- and sex-specific z-scores. Postnatal growth faltering (PGF) was defined as loss of >2 weight z-scores (severe) or simultaneous loss of >1 weight, length, and head circumference z-scores (complex).

**Results:**

The prevalence of severe and complex PGF was 11.7% and 22%, respectively. Infants <28 weeks’ gestation experienced more PGF (severe: 22.2%; complex: 33.3%) and feeding delays. Older age at first and full enteral feeding, and longer transition from first to full enteral feeding, were associated with slower growth (All, *p* < 0.05). Feeding delays were associated with prolonged hospitalization (*p* < 0.05).

**Conclusion:**

Feeding milestones and postnatal growth are interrelated. Earlier attainment of feeding milestones may support more favorable growth outcomes in preterm-born infants.

## Introduction

The provision of enteral nutrition to preterm infants (less than 32 weeks’ gestation) is challenging owing to their immature aerodigestive functions and gut motility, and they are at high risk for morbidities [1, 2]. Consequently, carefully structured progressing enteral feeding protocols are complemented with parenteral nutrition beginning at birth until gradually transitioned to full enteral feedings as the infant attains enteral feeding tolerance [3, 4]. Early enteral feedings begin with the provision of milk feeds (human milk or formula) as minimal enteral feeding within a few days of birth [3, 4]. This is followed by a gradual increase in milk volumes and a decrease in parenteral nutrition volumes until full enteral feeding is achieved and the infant is completely weaned off parenteral support [3]. Subsequently, oral feeding is initiated and progressed based on the infant’s oral motor cues, cardiorespiratory stability, and gastrointestinal tolerance [5].

Although the goal of this structured feeding protocol is to help achieve recommended nutrient intakes that support the growth of very preterm infants at a rate similar to that of a typical fetus of the same postmenstrual age (PMA), this is rarely achieved. At discharge from the hospital, a large proportion of these infants are at a significantly lower than expected percentile and z-score for weight, length, and head circumference [6, 7]. This faltering growth often seen in preterm infants is associated with short- and long-term morbidity and mortality, including adverse neurodevelopmental and cardiometabolic outcomes [8, 9]. While previous studies have reported that earlier initiation of enteral feeding and shorter progression to full enteral feeding are associated with better growth outcomes [10, 11], data are scant with regard to the associations between both enteral and oral feeding milestones and postnatal growth.

Specifically, it remains unclear whether the maturational age at first and full oral feeding, as well as the duration of transition from first to full oral feeds, are independently associated with postnatal growth. Prior studies have also not examined growth trajectory as a continuous variable in relation to feeding milestones. Furthermore, several comorbidities of prematurity, such as necrotizing enterocolitis (NEC), bronchopulmonary dysplasia (BPD), and sepsis, may influence both feeding progression and growth [12–14].

Therefore, the aim of this study was to examine the relationship between feeding milestones and comorbidities with the postnatal growth of preterm infants.

## Methods

### Study sample, design, and setting

This retrospective study examined data collected from infants referred to the Innovative Infant Feeding Disorders Program at the all-referral level IV NICU of Nationwide Children’s Hospital in Columbus, Ohio, between January 2011 and December 2023. Infants were referred while inpatients for concerns related to severe feeding and aerodigestive difficulties, including gastroesophageal reflux disease (GERD), feeding refusal, dysmotility, inadequate sucking ability relative to maturational age, feeding or postprandial cardiorespiratory complications, and pre-gastrostomy placement evaluation.

Inclusion criteria were those infants born at less than 32 weeks’ gestation, those with birth weight under 1500 g, and those discharged at a term-equivalent PMA (37–42 weeks). These criteria were selected because very preterm ( < 32 weeks’ gestation) and very low birthweight ( < 1500 g) infants are the greatest risk for postnatal growth faltering [6, 7]. The goal was to evaluate postnatal growth up to term gestation, hence only infants discharged at term-equivalent PMA were included. Growth data at term-equivalent PMA were unavailable if the infant was discharged at an earlier or later age.

Exclusion criteria included the presence of chromosomal or genetic disorders and inadequate documentation of growth measures and feeding milestone characteristics. The criteria to include infants discharged at term equivalent PMA resulted in a sample of infants discharged home on full oral feeds only. This was because infants requiring tube feedings at discharge were usually discharged at a later PMA in our setting.

A priori sample size was not set as this study was intended to be exploratory. Hence, all infants meeting the specified inclusion and exclusion criteria were included in the present study. Institutional Review Board approval was obtained, parental informed consent was obtained for all infants, and Health Insurance Portability and Accountability Act guidelines were adhered to.

### Data collection and analysis

Clinical characteristics were obtained from the electronic medical record (EPIC), a secure software used to store patient information, and managed using REDCap (Research Electronic Data Capture) [15]. Gestational age was calculated from the first day of the last menstrual period and confirmed by ultrasonography, when available. Postmenstrual age (PMA) at discharge was calculated as gestational age plus chronological age in weeks. Bronchopulmonary dysplasia (BPD) was defined as the need for supplementary respiratory support at 36 weeks PMA [16]. Necrotizing enterocolitis (NEC) was defined as Bell stage II or III [17]. Patent ductus arteriosus (PDA) was diagnosed using echocardiography and clinical criteria [18]. Intraventricular hemorrhage (IVH) was diagnosed with ultrasonography and defined using Papile’s classification as stages 1 to 4 [19]. Sepsis was either early ( < 72 h) or late onset ( ≥ 72 h) and was diagnosed based on a positive blood, urine, or cerebrospinal fluid culture [20].

Feeding data obtained from the medical records included the dates of the first enteral feeding, full enteral feeding, first oral feeding, and full oral feeding. The feeding milestones were defined as the age at which feeding skills were achieved. The enteral feeding milestones were the days of life at first, and full enteral feedings, while the oral feeding milestones were the PMA at first, and full oral feedings [21, 22]. First enteral feeding was defined as the first enteral offering, irrespective of the volume. Full enteral feeding was defined as achieving ≥120 mL/kg/day of enteral intake with complete discontinuation of parenteral nutrition. First oral feeding was defined as the first oral intake, irrespective of the volume. Full oral feeding was defined as the intake of all food orally without the need for a feeding tube for two consecutive days. Full enteral and full oral feeding volumes were set at 120 ml/kg/day because BPD is widespread in our setting, and most infants with BPD were on feeding volume restrictions [21]. Using our unit’s simplified, individualized, milestone-targeted, pragmatic, longitudinal, and educational (SIMPLE) feeding program criteria, targeted feeding milestones were defined as (a) start of first enteral feeding by the third day of life, (b) progression to full enteral feeding by the fourteenth day of life, (c) first oral feeding by 34 weeks PMA and (d) full oral feeding by 38 weeks PMA [21].

Growth data included weight, length, and head circumference at birth and at discharge, converted to age- and sex-specific z-scores using the Fenton growth charts [23]. Weight gain velocity was calculated as grams per kilogram per day (g/kg/day) by dividing the average daily weight gain (in grams) over the mean weight (in kilograms) during that time [24]. Linear and head growth rates were expressed as cm/week. Postnatal growth faltering (PGF) was defined using two criteria: (a) severe PGF at discharge, defined as a weight z-score decline from birth to discharge greater than two standard deviations [25], (b) complex PGF defined as a simultaneous decline of weight, length, and head circumference z-scores from birth to discharge greater than one standard deviation [13]. Growth trajectories for weight, length, and head circumference were defined as the change in z-scores from birth to discharge.

### Statistical analysis

Infant categories based on gestational age ( < 28 weeks and ≥28 weeks) and PGF status at discharge were compared using the Pearson chi-square and Fisher exact tests for categorical variables, and the t-test and Mann-Whitney U test (when the normality assumption was not met) for continuous variables. Effect sizes with 95% confidence intervals were calculated to complement statistical significance testing: Cohen’s d for continuous variables and odds ratios (OR) for categorical variables. Univariate linear regression was used to examine the relationship between growth trajectories (differences in weight, length, and head circumference z-scores from birth to discharge) and feeding milestones. Spearman’s rank correlation was also reported as a non-parametric measure that does not assume linearity or normality.

Multivariable models were developed using a multi-stage, systematic approach. First, univariate regression was performed for all candidate predictors identified based on prior literature and clinical relevance, with predictors showing *p* ≤ 0.10 retained for further analysis. Multivariate models were then constructed using forward selection (entry *p* = 0.25), stepwise selection (entry *p* = 0.35, stay *p* = 0.25), and Least Absolute Shrinkage and Selection Operator (LASSO) regression. Candidate models were compared using the Akaike Information Criterion (AIC), with the model exhibiting the lowest AIC value selected to strike a balance between goodness of fit and model parsimony. Collinearity among predictors in the selected models was assessed using the Condition Index and pairwise correlations. A Condition Index >30 indicates serious collinearity, while values between 10 and 30 suggest moderate collinearity.

Data are presented as numbers (percentages) for categorical variables, mean ± SD or median (IQR) for continuous variables, and regression estimates with 95% confidence intervals (CI). All tests were two-sided, and a *p*-value of <0.05 was considered statistically significant. Analyses were performed using SAS version 9.4 and IBM SPSS Statistics version 28.

## Results

A total of 1654 infants were referred to the Innovative Neonatal and Feeding Disorders Program between January 2011 and December 2023. Among them, 143 infants met all the study criteria for being very preterm, very low birth weight, and discharged at term-equivalent age. After excluding 9 infants with chromosomal disorders and 57 infants with incomplete documentation, the final sample included 77 infants. Clinical characteristics stratified by gestational age are presented in Table 1. Compared with very preterm infants ( ≥ 28 weeks), extremely preterm infants had significantly lower median APGAR scores at both 1 minute (*p* = 0.040) and 5 min (*p* = 0.002). They were also more likely to require mechanical ventilation at birth (OR = 0.06 [95% CI 0.02–0.21], *p* < 0.001) and respiratory support at discharge (OR = 0.19 [95% CI 0.07–0.57], *p* = 0.003). Extremely preterm infants were also at increased odds of having PDA (OR = 0.27 [95% CI 0.09–0.81], *p* = 0.020) and BPD (OR = 0.09 [95% CI 0.03–0.27], *p* < 0.001), and they spent significantly more days in the hospital (96.2 ± 13.8 vs 74.2 ± 11.6 days, *p* < 0.001).

**Table 1:** Clinical Characteristics of the Infants Stratified by Gestational Age.

Characteristic	Overall *n* = 77	<28 weeks GA *n* = 36	≥28 weeks GA *n* = 41	Effect (95% CI)	*P*-value
**At Birth**					
Male sex, *n* (%)	42 (54.5)	17 (47.2)	25 (61.0)	0.57 (0.23, 1.42)	0.258
Gestational age, weeks	28.2 ± 2.0	26.5 ± 1.4	29.6 ± 1.1	−2.62 (−3.32, −2.07)	<0.001
Apgar at 1 min, median (range)	4 (1–9)	3.5 (1–7)	5 (1–9)	−0.50 (−0.97, −0.05)	0.040
Apgar at 5 min, median (range)	7 (1–9)	7 (1–9)	8 (2–9)	−0.66 (−1.15, −0.22)	0.002
Mechanical ventilation at birth, *n* (%)	27 (35.1)	23 (63.9)	4 (9.9)	0.06 (0.02, 0.21)	<0.001
**At Discharge**					
Respiratory support at discharge, *n* (%)	23 (29.9)	17 (47.2)	6 (14.6)	0.19 (0.07, 0.57)	0.003
PMA at discharge, weeks	40.2 ± 1.3	40.2 ± 1.3	40.2 ± 1.2	−0.03 (−0.49, 0.42)	0.881
Length of hospital stay, days	84.5 ± 16.7	96.2 ± 13.8	74.2 ± 11.6	1.74 (1.25, 2.32)	<0.001
Any human milk feeding at discharge	23 (29.9)	10 (27.8)	13 (31.7)	1.21 (0.45, 3.22)	0.805
**Morbidities**					
PDA, *n* (%)	20 (26)	14 (38.9)	6 (14.6)	0.27 (0.09, 0.81)	0.020
IVH, *n* (%)	21 (27.3)	13 (36.1)	8 (19.5)	0.43 (0.15, 1.2)	0.128
Sepsis (Culture Positive), *n* (%)	12 (15.6)	8 (22.2)	4 (9.8)	0.38 (0.10, 1.38)	0.208
NEC, *n* (%)	6 (7.8)	4 (11.1)	2 (4.9)	0.41 (0.07, 2.39)	0.410
BPD, *n* (%)	36 (46.8)	27 (75.0)	9 (22.0)	0.09 (0.03, 0.27)	<0.001
**Feeding and Targeted Feeding Milestones**					
DOL at first EF, days	2.5 ± 4.5	4.8 ± 6.1	2.4 ± 1.9	0.55 (0.10, 1.03)	0.029
First EF by DOL 3, *n* (%)	62 (80.5)	25 (69.4)	37 (90.2)	4.07 (1.16, 14.23)	0.041
DOL at full EF, days	15.6 ± 10.6	22.0 ± 12.5	11.9 ± 5.2	1.08 (0.62, 1.60)	<0.001
Full EF by DOL 14, *n* (%)	42 (54.5)	10 (27.8)	32 (78.0)	9.24 (3.27, 26.12)	<0.001
First to full EF, days	13.1 ± 8.0	17.2 ± 8.4	9.5 ± 5.6	1.10 (0.64, 1.62)	<0.001
PMA at first PO, weeks	34.6 ± 1.4	35.1 ± 1.4	34.1 ± 1.1	0.76 (0.31, 1.26)	<0.001
First PO by 34 weeks PMA, *n* (%)	31 (40.3)	9 (25.0)	25 (61.0)	4.69 (1.76, 12.51)	0.003
PMA at full PO, weeks	37.4 ± 1.8	37.5 ± 1.7	37.3 ± 1.8	0.09 (−0.37, 0.54)	0.707
Full PO by 38 weeks PMA, *n* (%)	49 (63.6)	24 (66.7)	26 (63.4)	0.87 (0.34, 2.22)	0.814
First PO to full PO, days	20.0 ± 13.1	16.9 ± 12.8	22.7 ± 13.0	−0.44 (−0.92, 0.00)	0.055

*BPD* Bronchopulmonary dysplasia, *DOL* day of life, *EF* enteral feeding, *GA* gestational age, IVH intraventricular hemorrhage, *NEC* necrotizing enterocolitis, *PDA* patent ductus arteriosus, *PMA* postmenstrual age, *PO* per oral.

Data are presented as *n* (%), median (range), or mean ± SD. Reference group =≥28 weeks GA. The effect size column represents Cohen’s D for continuous variables and odds ratios for categorical data. All P-values were two-sided and were considered significant at *p* < 0.05.

Feeding progression differed markedly by gestational age as extremely preterm infants reached key enteral feeding milestones later than very preterm infants. They achieved first enteral feeding at a later age (4.8 ± 6.1 vs 2.4 ± 1.9 days, *p* = 0.029) and were less likely to receive any enteral feeds by the third day of life (OR = 4.07 [95% CI 1.16–14.23], *p* = 0.041). Time to full enteral feeding was also longer among extremely preterm infants (22.0 ± 12.5 vs 11.9 ± 5.2 days, *p* < 0.001), and they were less likely to achieve full enteral feeds by the fourteenth day of life (OR = 9.24 [95% CI 3.27–26.12], *p* < 0.001). Consistent with these findings, the interval from first to full enteral feeding was prolonged in the extremely preterm group (17.2 ± 8.4 vs 9.5 ± 5.6 days, *p* < 0.001). For oral feeding, extremely preterm infants reached first oral feeding at a later PMA (35.1 ± 1.4 vs 34.1 ± 1.1 weeks, *p* < 0.001) and were less likely to achieve this milestone by 34 weeks PMA (OR = 4.69 [95% CI 1.76–12.51], *p* = 0.003). However, the timing of full oral feeding, the likelihood of achieving full oral feeds by 38 weeks PMA, and the interval from first to full oral feeding did not differ significantly between groups (*p* > 0.05).

The growth characteristics of the infants are presented in Table 2. More than one-third of the cohort (35.1%) had extremely low birth weight ( < 1000 g), and extremely preterm infants were far more likely to fall into this category (63.9% vs 9.8%; OR = 0.06 [95% CI 0.02–0.21], *p* < 0.001). Only three infants (3.9%) were small for gestational age at birth, defined as a birthweight percentile <10%. By discharge, however, 31.2% of infants had a weight percentile <10%, which is indicative of substantial postnatal growth faltering. A similar pattern was observed for length: 11.7% were <10th percentile at birth, and this increased significantly to 57.1% at discharge. Weight, length, and head circumference z-scores were also significantly higher at birth than at discharge, a reflection of consistent downward growth trajectories during hospitalization. Extremely preterm infants experienced greater declines in weight, length, and head circumference z-scores from birth to discharge than very preterm infants (all *p* < 0.05). Average weight gain velocity was 11.5 ± 1.3 g/kg/day, and no infant achieved the commonly targeted 15 g/kg/day. Severe and complex postnatal growth faltering occurred in 11.7% and 22% of infants, respectively. Extremely preterm infants were at greater odds for experiencing either severe or complex growth faltering (severe PGF: OR = 11.36 [95% CI 1.35–100], *p* = 0.010; complex PGF: OR = 3.60 [95% CI 1.12–11.49], *p* = 0.031).

**Table 2:** Growth Characteristics of the Infants Stratified by Gestational Age.

Growth Characteristics	Overall *n* = 77	<28 weeks GA *n* = 36	≥28 weeks GA *n* = 41	Effect (95% CI)	*P*-value
**Weight**					
Birth weight, g	1094.6 ± 225.2	970.5 ± 210.4	1203.5 ± 177.9	−1.21 (−1.73, −0.74)	<0.001
Birth weight < 1000 g, *n* (%)	27 (35.1)	23 (63.9)	4 (9.8)	0.06 (0.02, 0.21)	<0.001
Birth weight z-scores	0.1 ± 0.9 ^a^	0.6 ± 0.9	–0.3 ± 0.8	1.03 (0.58, 1.55)	<0.001
Birth weight percentile < 10%, *n* (%)	3 (3.9) ^b^	1 (2.8)	2 (4.9)	1.80 (0.16, 20.83)	1.000
Discharge weight, g	3143.0 ± 481.2	3207.6 ± 482.9	3087.9 ± 478.6	0.25 (−0.20, 0.71)	0.279
Discharge weight z-scores	−0.9 ± 0.9 ^c^	−0.7 ± 0.9	−1.1 ± 0.9	0.38 (−0.07, 0.85)	0.097
Discharge weight percentile <10%, *n* (%)	24 (31.2) ^d^	8 (22.2)	16 (39)	0.45 (0.16, 1.22)	0.142
Weight gain velocity (g/kg/day)	11.5 ± 1.3	11.2 ± 1.1	11.8 ± 1.3	−0.47 (−0.95, −0.02)	0.041
Weight z-score difference from birth to discharge	−1.0 ± 0.8	−1.3 ± 0.9	−0.8 ± 0.7	−0.66 (−1.15, −0.21)	0.006
Severe PGF (Weight z-score decline > 2), *n* (%)	9 (11.7)	8 (22.2)	1 (2.4)	11.36 (1.35–100)	0.010
***Length***					
Birth length, cm	36.2 ± 2.8	34.6 ± 2.9	37.7 ± 1.8	−1.32 (−1.86, −0.85)	<0.001
Birth length z-scores	0.0 ± 1.0 ^e^	0.4 ± 1.07	−0.3 ± 0.9	0.70 (0.25, 1.19)	0.004
Birth length percentile < 10%, *n* (%)	9 (11.7) ^f^	4 (11.1)	5 (12.2)	0.90 (0.22, 3.65)	0.630
Discharge length, cm	47.8 ± 2.3	48.1 ± 2.4	47.5 ± 2.2	0.23 (-0.22, 0.70)	0.312
Discharge length z-scores	−1.4 ± 1.0 ^g^	−1.3 ± 0.9	−1.6 ± 1.0	0.32 (−1.13, 0.79)	0.163
Discharge length percentile <10%, n (%)	44 (57.1) ^h^	19 (52.8)	25 (61)	0.72 (0.29, 1.77)	0.497
Length gain velocity, cm/week	1.0 ± 0.2	1.0 ± 0.1	0.9 ± 0.2	0.37 (−0.08, 0.84)	0.107
Length z-score difference from birth to discharge	−1.5 ± 0.9	−1.7 ± 1.0	−1.3 ± 0.8	−0.43 (−0.90, 0.02)	0.069
***Head Circumference (HC)***					
Birth HC, cm	25.5 ± 1.8	24.2 ± 1.7	26.7 ± 1.0	−1.89 (−2.39, −1.31)	<0.001
Birth HC z-scores	0.0 ± 0.9 ^i^	0.3 ± 0.9	−0.2 ± 0.8	0.55 (0.24, 1.03)	0.019
Birth HC percentile < 10%, *n* (%)	0 (0)	0 (0)	0 (0)	–	–
Discharge HC, cm	34.1 ± 1.4	34.1 ± 1.5	34.1 ± 1.4	−0.01 (−0.46, 0.45)	0.975
Discharge HC z-scores	−0.6 ± 0.8 ^j^	−0.6 ± 0.9	−0.6 ± 0.8	0.06 (−0.40, 0.51)	0.805
Discharge HC percentile <10%, *n* (%)	5 (6.5)	1 (2.8)	4 (9.8)	0.26 (0.03, 2.48)	0.364
HC gain velocity, cm/week	0.7 ± 0.1	0.7 ± 0.1	0.7 ± 0.1	0.19 (−0.26, 0.65)	0.402
HC z-score difference from birth to discharge	−0.6 ± 0.9	−0.8 ± 1.0	−0.4 ± 0.8	−0.45 (−0.92, −0.00)	0.056
Complex Postnatal Growth Faltering	17 (22)	12 (33.3)	5 (12.2)	3.60 (1.12, 11.49)	0.031

*HC* head circumference.

Data are presented as *n* (%) or mean ± SD. a vs c, b vs d, e vs g, f vs h, and i vs j were significantly different (*P* < 0.001). Reference group =≥28 weeks GA. The effect size column represents Cohen’s D for continuous variables and odds ratios for categorical data. All P-values were two-sided and were considered significant at *p* < 0.05.

Table 3 presents the comparison of clinical characteristics of the infants, stratified by severe or complex PGF status at discharge. Gestational age was lower, the need for mechanical ventilation at birth was higher, and the odds of sepsis were higher among infants with either severe or complex PGF (all *p* < 0.05). However, only infants with severe PGF had significantly lower APGAR scores at 5 minutes, a higher odds of requiring respiratory support at discharge, and a longer length of hospitalization (all *p* < 0.05). Feeding progression was delayed in both groups: however infants with either severe and complex PGF were less likely to achieve early enteral feeding by the third day of life (severe PGF: OR = 0.14 [95% CI 0.03–0.60], *p* = 0.012; complex PGF: OR = 0.22 [95% CI 0.07–0.74], *p* = 0.017), reached full enteral feeds at a later age (severe PGF: 25.6 ± 8.6 (PGF) vs 15.4 ± 10.3 (non PGF) DOL, *p* = 0.007; complex PGF: 22.8 ± 12.9 (PGF) vs 14.8 ± 9.2 (non PGF) DOL, *p* = 0.028), and were less likely to achieve full enteral feeds by the fourteenth day of life (complex: OR = 0.26 [95% CI 0.08–0.83], *p* = 0.027). The number of days from first to full enteral feeding was also significantly longer in infants with either severe or complex PGF (severe PGF: 21.3 ± 2.6 (PGF) vs 12.0 ± 7.4 (non PGF) days, *p* = 0.006; complex PGF: 18.1 ± 8.8 (PGF) vs 11.7 ± 7.2 (non PGF) days, *p* = 0.011). Oral feeding milestones and progression did not differ significantly between the groups (all *p* > 0.05).

**Table 3:** Factors Associated with Severe and Complex Postnatal Growth Faltering (Unadjusted).

	Severe				Complex			
	PGF *n* = 9	Non PGF *n* = 68	Effect (95% CI)	*P*-value	PGF *n* = 17	Non PGF *n* = 60	Effect (95% CI)	*P-*value
**At birth**								
Male sex, *n* (%)	3 (33.3)	39 (57.4)	0.37 (0.09, 1.61)	0.286	9 (52.9)	33 (55)	0.92 (0.31, 2.70)	1.000
Gestational age, weeks	25.5 ± 2.3	28.5 ± 1.7	−1.73 (−2.54, 1.02)	<0.001	26.8 ± 2.4	28.5 ± 1.7	−0.93 (−1.53, −0.39)	0.011
Gestational age < 28 weeks	8 (88.9)	28 (41.2)	11.4 (1.35–96.58)	0.010	12 (70.6)	24 (40)	3.60 (1.12, 11.53)	0.031
Apgar at 1 min, median (range)	3 (1–5)	5 (1–9)	−0.62 (−1.35, 0.08)	0.087	4 (1–7)	4 (1–9)	−0.18 (−0.74, 0.36)	0.855
Apgar at 5 min, median (range)	5 (1–7)	8 (2–9)	−1.56 (−2.36, −0.85)	0.008	7 (1–9)	8 (2–9)	−0.51 (−1.08, 0.02)	0.178
Mechanical ventilation, *n* (%)	8 (88.9)	19 (27.9)	20.62 (2.41, 176.12)	<0.001	11 (64.7)	16 (26.7)	5.04 (1.6, 15.88)	0.008
**At discharge**								
Respiratory support at DC, *n* (%)	6 (66.7)	17 (25)	6.0 (1.35, 26.64)	0.018	8 (47.1)	15 (25)	2.67 (0.87, 8.15)	0.131
PMA at discharge, weeks	40.2 ± 1.6	40.2 ± 1.2	−0.03 (−0.74, 0.67)	0.939	40.0 ± 1.3	40.3 ± 1.2	−0.27 (−0.82, 0.27)	0.368
Length of hospital stay, days	103.0 ± 19.3	82.0 ± 14.9	1.36 (0.66, 2.14)	0.012	92.2 ± 21.3	82.3 ± 14.7	0.61 (0.07, 1.18)	0.085
Human milk feeding at DC, *n* (%)	0 (0.0)	23 (33.8)	–	–	5 (29.4)	18 (30)	0.97 (0.30, 3.17)	1.000
**Morbidities**								
PDA, *n* (%)	4 (44.4)	16 (23.5)	2.6 (0.62, 10.86)	0.227	7 (41.2)	13 (21.7)	2.53 (0.81, 7.95)	0.125
IVH, *n* (%)	5 (55.6)	16 (23.5)	4.06 (0.97, 16.96)	0.057	7 (41.2)	14 (23.3)	2.3 (0.74, 7.16)	0.216
Sepsis (Culture Positive), *n* (%)	5 (55.6)	7 (10.3)	10.89 (2.36, 50.29)	0.004	6 (35.3)	6 (10)	4.91 (1.33, 18.09)	0.020
NEC, *n* (%)	1 (11.1)	5 (7.4)	1.58 (0.16, 15.24)	0.538	2 (11.8)	4 (6.7)	1.87 (0.21, 11.19)	0.608
BPD, *n* (%)	7 (77.8)	29 (42.6)	4.71 (0.91, 24.35)	0.074	10 (58.8)	26 (43.3)	1.87 (0.63, 5.57)	0.284
**Feeding**								
DOL at first EF, days	4.2 ± 2.3	3.4 ± 4.8	0.18 (-0.52, 0.89)	0.405	4.7 ± 5.7	3.2 ± 4.2	0.33 (-0.21, 0.89)	0.328
First EF by DOL 3, *n* (%)	4 (44.4)	58 (85.3)	0.14 (0.03, 0.60)	0.012	10 (58.8)	52 (86.7)	0.22 (0.07, 0.74)	0.017
DOL at full EF, days	25.6 ± 8.6	15.4 ± 10.3	1.00 (0.31, 1.76)	0.007	22.8 ± 12.9	14.8 ± 9.2	0.78 (0.24, 1.36)	0.028
Full EF by DOL 14, *n* (%)	2 (22.2)	40 (58.8)	0.2 (0.04, 1.04)	0.071	5 (29.4)	37 (61.7)	0.26 (0.08, 0.83)	0.027
First to full EF, days	21.3 ± 2.6	12.0 ± 7.4	1.25 (0.55, 2.03)	0.006	18.1 ± 8.8	11.7 ± 7.2	0.85 (0.31, 1.44)	0.011
PMA at first PO, weeks	34.6 ± 1.8	34.5 ± 1.3	0.08 (-0.63, 0.79)	0.866	34.7 ± 1.6	34.5 ± 1.3	0.12 (-0.4, 0.67)	0.704
First PO by 34 weeks PMA, *n* (%)	3 (33.3)	31 (45.6)	0.60 (0.14, 2.59)	0.723	6 (35.3)	28 (46.7)	0.62 (0.20, 1.90)	0.581
PMA at full PO, weeks	36.9 ± 1.8	37.5 ± 1.7	−0.34 (−1.05, 0.36)	0.385	37.0 ± 1.4	37.5 ± 1.8	−0.32 (−0.88, 0.22)	0.172
Full PO by 38 weeks PMA, *n* (%)	7 (77.8)	43 (63.2)	2.04 (0.39, 10.56)	0.481	14 (82.4)	36 (60)	3.11 (0.81, 11.99)	0.148
First PO to full PO, days	15.7 ± 12.4	20.5 ± 13.2	−0.37 (−1.09, 0.33)	0.295	16 ± 12.3	21.1 ± 13.3	−0.39 (−0.95, 0.14)	0.148

*BPD* Bronchopulmonary dysplasia, *DOL* day of life, *EF* enteral feeding, *IVH* intraventricular hemorrhage, *NEC* necrotizing enterocolitis, *PDA* patent ductus arteriosus, *PGF* postnatal growth faltering, *PMA* postmenstrual age, *PO* per oral.

Data are presented as *n* (%), median (range), or mean ± SD. Effect size column represents Cohen’s D for continuous variables and odds ratios for categorical data. All P-values were two-sided and were considered significant at *p* < 0.05.

**Fig. 1:**
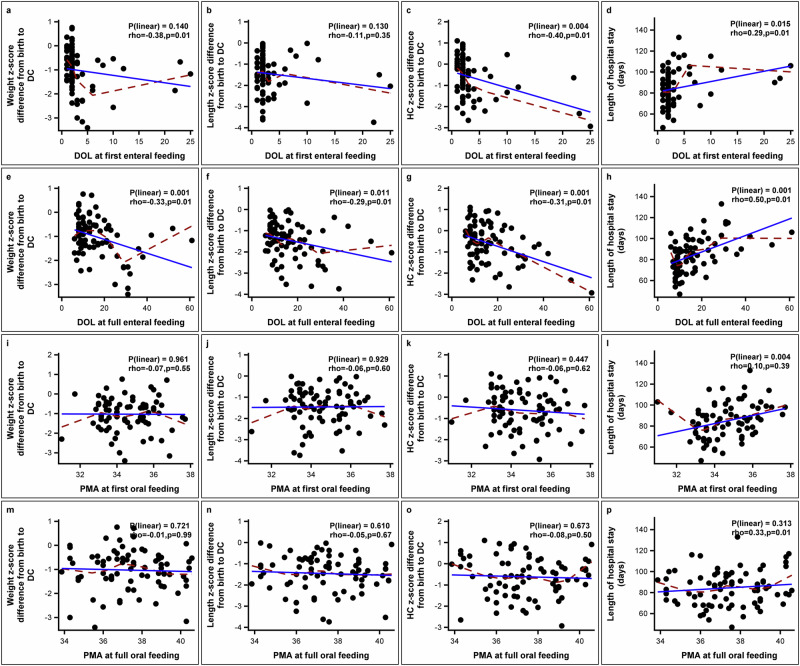
Relationship between feeding milestones, growth z-score differences from birth to discharge, and the length of hospital stay. Day of life (DOL) at first enteral feeding was negatively associated with weight (**a**) and head circumference (**c**) z score change from birth to discharge, not associated with length z score change (**b**), and positively associated with length of hospital stay (**d**). DOL at full enteral feeding was negatively associated with weight (**e**), length (**f**), and head circumference (**g**) z score change, and positively associated with length of hospital stay (**h**). Postmenstrual age (PMA) at first oral feeding was not associated with weight (**i**), length (**j**), or head circumference (**k**) z score change, but was positively associated with length of hospital stay (**l**). PMA at full oral feeding was not associated with weight (**m**), length (**n**), or head circumference (**o**) z score change, but was positively associated with length of hospital stay (**p**). In the figure, observed values are represented by dots, the bold lines are the regression line, and the dashed lines represent the B-spline lines.

Figure 1 shows the unadjusted relationship between feeding milestones and the weight, length, and head circumference trajectory from birth to discharge. The day of life at first enteral feeding was negatively correlated with weight (*ρ* = −0.38, *p* = 0.01) and head circumference difference from birth to discharge (*ρ* = −0.40, *p* = 0.01), while the day of life at full enteral feeding was negatively associated with weight (*ρ* = −0.33, *p* = 0.01), length (*ρ* = −0.29, *p* = 0.01), and head circumference (*ρ* = −0.31, *p* = 0.01) z-score differences from birth to discharge. In contrast, no associations were found between growth trajectories and oral feeding milestones (all *p* > 0.05). Feeding delays were also associated with a longer length of hospital stay: Day of life at first enteral feeding (*ρ* = 0.29, *p* = 0.01), and at full enteral feeding (*ρ* = 0.50, *p* = 0.01), as well as PMA at first oral feeding (*ρ* = 0.33, *p* = 0.01) were each positively correlated with the length of hospital stay.

Table 4 presents the factors independently associated with weight, length, and head circumference growth trajectories from birth to discharge in multivariable analyses. Slower advancement from first to full enteral feeding was associated with a poorer weight z-score trajectory (−0.012 [95% CI −0.025-0.000] units per additional day, *p* = 0.050). Later initiation of enteral feeding was, however, associated with higher weight growth (0.034 [95% CI 0.008–0.060] units per additional day, *p* = 0.001), whereas achieving first enteral feeding by the third day of life was associated with a better weight trajectory (0.487 [95% CI 0.181–0.792] units higher compared with infants receiving first enteral feeding after third day of life, *p* = 0.002). Later initiation of enteral feeding was associated with a poorer length trajectory (− 0.016 [95% CI−0.029 to −0.002] units per additional day, *p* = 0.025), and a longer interval from first to full oral feeding was similarly associated with poorer linear growth (−0.009 [95% CI−0.015 to −0.003] units per additional day, *p* = 0.003). Later achievement of full enteral feeding was associated with a poorer head circumference trajectory (−0.007 [95% CI−0.013 to −0.001] units per additional day, *p* = 0.018). Infants with IVH had significantly poorer head growth (−0.118 [95% CI−0.235 to −0.001] units lower compared to those without IVH, *p* = 0.048), and female infants also demonstrated lower head circumference growth (−0.148 [95% CI−0.250 to −0.045] units lower compared to male infants, *p* = 0.005).

**Table 4:** Factors Associated with Weight, Length, and Head Circumference Growth Trajectory from Birth to Discharge.

	**Effect (95% CI)**	***P***-**value**
**Weight**		
First to full EF, days	−0.012 (−0.025, 0.000)	0.050
DOL at first EF, days	0.034 (0.008, 0.060)	0.001
First EF by DOL 3	0.487 (0.181, 0.792)	0.002
Birth length, cm	0.075 (0.038, 0.111)	<0.000
Weight gain velocity, g/kg/day	0.492 (0.422, 0.562)	<0.000
Length gain velocity, cm/week	1.244 (0.656, 1.832)	<0.000
PMA at discharge, weeks	0.220 (0.154, 0.286)	<0.000
**Length**		
Female Sex	−0.039 (−0.252,0.175)	0.723
Gestational age	0.226 (0.192, 0.260)	<0.000
Apgar at 5 min	−0.030 (−0.063, 0.002)	0.068
Length Gain Velocity, cm/week	4.970 (4.574, 5.367)	<0.000
DOL at First EF, days	−0.016 (−0.029, −0.000)	0.025
First PO to Full PO, days	−0.009 (−0.015, −0.003)	0.0034
First PO to full PO+ Female sex interaction	0.011 (0.002, 0.020)	0.019
**Head Circumference**		
Gestational age, weeks	0.139 (0.109, 0.170)	<0.000
Head gain velocity, cm/week	7.823 (7.330, 8.316)	<0.000
DOL at full EF, days	−0.007 (−0.013, −0.001)	0.018
IVH	−0.118 (−0.235,−0.001)	0.048
Female Sex	−0.148 (−0.250, −0.045)	0.005
Human Milk feeding at discharge	−0.150 (−0.259,−0.040)	0.007

*DOL* day of life, *EF* enteral feeding, *IVH* intraventricular hemorrhage, *PMA* postmenstrual age, *PO* per oral. Data are presented as estimates and their corresponding 95% confidence intervals. All P-values were two-sided and were considered significant at *p* < 0.05.

## Discussion

The present study demonstrated that postnatal growth faltering (PGF) is a significant problem among preterm infants with feeding difficulties. A recent study reported a similar prevalence among preterm infants, further highlighting the challenges preterm populations face in meeting adequate postnatal growth [13]. Lower gestational age was associated with both severe and complex PGF and independently predicted poorer length and head circumference trajectories. This suggests that immaturity at birth contributes substantially to adverse growth outcomes, and this may be related to the difficulties faced with adapting to postnatal life. Infants with PGF also had lower Apgar scores and were more likely to require mechanical ventilation at birth. These findings likely reflect greater early physiological instability, increased metabolic stress, and delayed stabilization, factors known to interfere with early nutrient delivery and growth [26, 27]. Importantly, multiple studies have shown that early postnatal declines in weight, length, and head circumference z-scores are associated with adverse neurodevelopmental outcomes, and this highlights the need for early identification of at-risk infants to ensure timely interventions aimed at optimizing their nutritional and medical management [28–31].

Multiple morbidities, such as sepsis, NEC, PDA, and BPD, are known to be associated with growth outcomes [13, 32–35]. The association of these morbidities with growth may be explained by inflammatory processes, which increase energy expenditure and metabolic demands, as well as through alteration of nutrient utilization and accretion [32, 33]. Additionally, morbidities or their treatments can lead to disruptions in nutrient administration due to feeding intolerance and fluid restrictions, or nutrient losses resulting from a lack of conservation mechanisms, thus resulting in inadequate nutrient accretion. [32, 33]. Furthermore, medications used for the treatment of various morbidities may have negative or catabolic effects on growth [36, 37]. In the present study, the incidence of all morbidities was higher in infants with either severe or complex PGF; however, only sepsis was at a significantly higher rate. IVH was also identified as an independent factor associated with poor head circumference growth trajectory. Studies have reported that both IVH and poor head growth are associated with adverse neurodevelopmental outcomes, with the former playing a stronger role [31]. Hence, poor head growth may be playing a mediating role in the relationship between IVH and poor neurodevelopment.

Enteral feeding practices play a critical role in the growth, nutrient intake, and postnatal adaptation of preterm-born infants. Full enteral feeding is typically achieved by the 14th day of life [21, 38]. In this study, a substantial proportion of infants, however, did not attain full enteral feeding by that time, and the transition period was longer than reported in previous studies, reflecting the profound feeding difficulties characteristic of this population [11, 38, 39]. Previous work has shown that earlier initiation and faster progression of enteral feeding are associated with more rapid postnatal weight gain, consistent with the broader relationship between feeding milestones and the postnatal growth of preterm infants [40]. Another study reported that infants who required a longer transition to full enteral feeding had significantly poorer weight and head circumference growth from birth to term-equivalent age [11]. Previous studies have identified the transition period, when the infant is weaned off parenteral nutrition with gradual increases in enteral feedings, as a particularly vulnerable period for substantial energy and nutrient deficits to occur [41, 42].

The observed discrepancy between the beneficial association of achieving first enteral feeding by the third day and the positive association between later day of life at first enteral feeding and weight gain in the current study likely reflects the distinct clinical information captured by these variables. While the third day of life milestone likely captured the most stable infants with better growth potential, the continuous timing variable included infants across the full range of initiation days and therefore reflects more heterogeneous patterns of illness severity, feeding tolerance, and clinical decision-making. It is plausible that infants who started enteral feeding later received more proactive parenteral nutrition, potentially offsetting early nutritional deficits and contributing to the small gains in weight observed in this study. In contrast, later initiation of enteral feeding was consistently associated with poorer linear growth, suggesting that length may be more sensitive to early nutritional delays and less amenable to short-term compensation through parenteral nutrition.

These observational findings collectively provide evidence to support the importance of transitioning to full enteral feeding earlier for optimal growth. However, clinical trials evaluating modifications to enteral feeding strategies have yielded inconsistent results [38, 43], contributing to ongoing variability in feeding practice [4, 44]. Consistent with our results, other studies have identified enteral feeding milestones as significant predictors of the length of hospital stay [21, 22]. Delayed feeding progression may reflect underlying illness severity and contribute to prolonged hospitalization through persistent nutritional deficits and delayed discharge readiness. Given the economic and psychosocial burden of extended hospital stays, early attainment of enteral feeding milestones is advantageous and should be prioritized when clinically feasible.

The American Academy of Pediatrics recognizes independent, safe oral feeding as an essential criterion for the hospital discharge of preterm infants [45]. However, achieving oral feeding competence requires maturation of aerodigestive, enteric, and central nervous system functions, as well as consistent implementation of oral feeding practices. Evidence-based guidelines for oral feeding initiation and progression remain limited, contributing to substantial practice variation [46]. Although concerns exist that inefficient or uncoordinated oral feeding may increase energy expenditure and impair growth, oral feeding milestones were not consistently associated with growth trajectories in the present study. Similar findings have been reported elsewhere, with one trial showing that early initiation or rapid advancement of oral feeding did not affect weight gain [47]. The acquisition of comparable oral feeding milestones in all infants suggests that the appropriate maturation of airway and digestive functions was present [48]. Intensive inpatient use of proactive oral feeding strategies may have resulted in fewer observed differences despite the PGF [21, 22]. Additionally, at the time of oral feeding initiation and progression, infants are in a stable phase, with most morbidities having been resolved, which may also explain the lack of association with growth outcomes.

Like any retrospective study, this study has some limitations. The sample was drawn from a highly specialized population of preterm infants with profound feeding difficulties and included only those who met the strict selection criteria. As a result, the sample size was small, the potential for residual confounding is high, and the findings may not be generalizable to broader preterm infant populations. Data was collected over a 12-year period, during which clinical practices and nutrition protocols may have evolved, potentially influencing growth and feeding outcomes. Specific data on nutritional compositions and practices were not available, limiting our ability to examine the contribution of nutrient intakes. Additionally, formal adjustments for multiple comparisons were not made, and some confidence intervals were wide, reflecting limited statistical power for certain associations and underscoring the need for larger studies to confirm these findings.

Nonetheless, the study had several important strengths. We used a longitudinal classification of growth faltering that is considered more reflective of adverse growth patterns and malnutrition [25, 49]. Growth was also assessed as a continuous variable, avoiding the arbitrary dichotomization inherent in classifying infants as growth-faltered or not. Additionally, our sample included a population of infants with profound feeding difficulties during their NICU course, whose growth outcomes have not been explicitly studied. Together, these strengths position the study as an important foundation for future work aimed at refining feeding practices, improving early identification of infants at risk for postnatal growth faltering, and developing targeted nutritional strategies to support optimal growth and neurodevelopment in preterm infants with complex feeding challenges.

## Conclusion

In summary, postnatal growth faltering is common among preterm infants with feeding difficulties and is associated with birth characteristics, neonatal morbidities, and enteral feeding milestones. Lower gestational age, early physiologic instability, and sepsis are significant contributors to impaired growth, while intraventricular hemorrhage independently predicts slower head circumference trajectories. Delays in achieving enteral feeding milestones are consistently linked to poorer growth outcomes and prolonged hospitalization, emphasizing the importance of timely advancement of enteral nutrition. In contrast, oral feeding milestones are not strongly associated with growth, likely due to the more stable clinical phase during which oral feeding is initiated and the supportive feeding practices implemented in the neonatal intensive care unit. These findings underscore the need for early identification of infants at risk for suboptimal growth and for targeted strategies that optimize enteral feeding progression to support both growth and timely discharge. Continued implementation of proactive oral feeding strategies is recommended to support discharge readiness.

## Data Availability

The datasets generated during and/or analyzed during the current study are not publicly available due to privacy and ethical restrictions, but are available from the corresponding author on reasonable request.
